# Clinical and economic impact of COVID-19 on people with obesity in a Spanish cohort during the first pandemic peak

**DOI:** 10.3389/fendo.2023.1146517

**Published:** 2023-05-26

**Authors:** Torrego-Ellacuría M, Rubio-Herrera MA, González López-Valcárcel B, Fuentes-Ferrer ME, Martín V, Poyato F, Barber-Pérez P, Santucci C, Nuñez A, González-Pérez C, Luaces M

**Affiliations:** ^1^ Innovation Unit, Hospital Clínico San Carlos, Instituto de Investigación Sanitaria del Hospital Clínico San Carlos (IdISSC), Madrid, Spain; ^2^ Department of Endocrinology and Nutrition, Hospital Clínico San Carlos, Instituto de Investigación Sanitaria del Hospital Clínico San Carlos (IdISSC), Madrid, Spain; ^3^ Department of Medicine, Faculty of Medicine, Universidad Complutense de Madrid, Madrid, Spain; ^4^ Department of Quantitative Methods for Economics and Management, Universidad de Las Palmas de Gran Canaria, Las Palmas de Gran Canaria, Spain; ^5^ Department of Preventive Medicine, Hospital Clínico San Carlos, Instituto de Investigación Sanitaria del Hospital Clínico San Carlos (IdISSC), Madrid, Spain; ^6^ Research Unit, Hospital Universitario Nuestra Señora de Candelaria, Santa Cruz de Tenerife, Spain; ^7^ Department of Market Access and Public Affairs, Novo Nordisk Pharma, Sociedad Anónima (SA.), Madrid, Spain; ^8^ Department of Clinical, Medical and Regulatory, Novo Nordisk Pharma, Sociedad Anònima (SA.), Madrid, Spain; ^9^ Department of Clinical Sciences and Community Health, Università degli Studi di Milano, Milan, Italy; ^10^ Department of Critical Care, Hospital Clínico San Carlos, Instituto de Investigación Sanitaria del Hospital Clínico San Carlos (IdISSC), Madrid, Spain; ^11^ Department of Hospital Pharmacy, Hospital Clínico San Carlos, Instituto de Investigaciòn Sanitaria del Hospital Clínico San Carlos (IdISSC), Madrid, Spain

**Keywords:** COVID-19, obesity, invasive mechanical ventilation (IMV), intensive care unit (ICU), economic burden, obesity comorbidities, diabetes mellitus

## Abstract

**Introduction:**

COVID-19 and obesity relationship has been extensively studied since the COVID-19 outbreak, proving obesity is a risk factor. This study aims to broaden the available information about this association and to evaluate the economic impact of obesity and the COVID-19 disease combination.

**Methods:**

This retrospective study analyzed a sample of 3,402 patients admitted to a Spanish hospital with available body mass index (BMI) data.

**Results:**

The prevalence of obesity was 33.4%. Patients with obesity showed a higher risk of hospitalization (OR 95% ConfidenceInterval [CI]=1.46; [1.24-1.73]; *p* < 0.001), which increased with the obesity degree (I: OR [95% CI]=1.28 [1.06-1.55], *p* =0.010; II: OR [95% CI]=1.58 [1.16-2.15], *p* =0.004; III: OR [95% CI] =2.09 [1.31-3.34], *p* =0.002). Patients with type III obesity had a significantly higher risk of intensive care unit (ICU) admission (OR [95% CI]= 3.30 [1.67-6.53]; *p* = 0.001) and invasive mechanical ventilation (IMV) need (OR [95% CI]= 3.98 [2.00-7.94]; *p*<0.001). The average cost per patient was remarkably higher in patients with obesity (*p* = 0.007), reaching an excess cost of 28.41% in the study cohort and rising to 56.5% in patients < 70 years. The average cost per patient increased significantly with the degree of obesity (*p* = 0.007).

**Discussion:**

In conclusion, our results suggest a strong association between obesity and adverse COVID-19 outcomes and higher expenditures in patients with both conditions.

## 1 Introduction

The coronavirus disease (COVID-19) pandemic has been the most notable global concern of this century. Since its outbreak at the end of 2019, researchers worldwide have made an enormous effort to investigate and draw a picture not only of the disease itself but also of its association with other common diseases. COVID-19 is a respiratory illness caused by infection with severe acute respiratory syndrome coronavirus (SARS-CoV-2). It may present as an asymptomatic, mild, moderate, or severe disease, depending on the patient baseline condition among other factors.

On the other hand, obesity is a metabolic, systemic, chronic, multifactorial disease leading to increased subcutaneous and visceral adipose tissue. It is well known that obesity promotes inflammation and it is associated with the appearance of comorbidities such as diabetes mellitus (DM), arterial hypertension (HT), and/or cardiovascular disease (CVD), strongly related with mortality in the general population (1, 2). Nevertheless, some authors have concluded that not all obese patients have a deteriorated metabolic profile, and they have described different obesity phenotypes referring to their metabolic status independent on the person weight; Metabolically Healthy Obesity (MHO) and Metabolically Obese Normal Weight (MONW). These findings received the name of “obesity paradox”, which is still controversial and debate-generating (3–5). Obesity is present in approximately 13% of adults worldwide, and it continues to rise globally (6). The prevalence of obesity in the Spanish population is worryingly higher, rising to 21.6%, according to the ENPE study published in 2016 (7). The association between obesity and inflammation is well established, as the excess nutrients lead to activation of a metabolic signaling pathway which ends up causing activation of cytokines resulting in a low-grade inflammatory response (8). Clinical evidence has demonstrated that COVID-19 patients express a high level of cytokines, known as “cytokine storm”, and present hyperinflammation, which could be the link between COVID-19 and obesity (9). Obesity already plays a significant role in the hospitalization and mortality rate in other respiratory diseases caused by viral infections, such as other coronaviruses pandemics, SARS and MERS in 2002 and 2012, respectively, or the H1N1 flu pandemic in 2009 (10, 11).

The first published data following the COVID-19 outbreak came from China and the United States, suggesting that age, male sex, obesity, HT, CVD, DM, and chronic kidney disease were factors associated with COVID-19 adverse outcomes (12–14). By 2023, the relationship between obesity and other chronic diseases and COVID-19 has been highly studied. Obesity has been determined to be a risk factor for COVID-19, and it is associated with adverse outcomes in terms of higher rates of hospital admission, intensive care unit (ICU) admission, and need for invasive mechanical ventilation (IMV) (15–17). Moreover, the prevalence of obesity seems to be higher among patients developing acute respiratory distress syndrome (ADRS) (16, 18, 19). The association between obesity and higher mortality in COVID-19 patients is still controversial, with clear evidence being found in many studies (12, 17, 20–25); but lacking a reliable association between both conditions in other investigations (15, 18, 26).

The high rates of hospitalization, ICU admission, and need for IMV occurring during the COVID-19 pandemic, added to the cost of antiviral and coadjuvant drugs, entail an enormous impact on public health expenditure (27). Clearly, these expenses may be higher if patients present other underlying diseases besides COVID-19. The presence of obesity as one of the most prevalent chronic diseases in western countries, and specifically in Spain, and the critical impact seen in Spain during the peak of the pandemic, led us to investigate the use of resources in patients with overweight and obesity, obtaining clear results about the excess cost derived from obesity (28, 29).

This study aimed to assess the clinical and economic impact of the COVID-19 disease in patients diagnosed with obesity alone or obesity along with three specific obesity-related comorbidities: DM, HT and CVD in a Spanish hospital. According to the reviewed literature and the clinicians expertise, we hypothesized the impact of the disease combination to be significantly higher than the impact of COVID-19 infection itself.

## 2 Materials and methods

### 2.1 Study design and patients

The OBESITY-COVID study is a retrospective, registry-type study including COVID-19 positive patients admitted to Hospital Clínico San Carlos in Madrid (Spain) during the first wave of the COVID-19 pandemic (from March 1, 2020 to June 30, 2020). The study was conducted in accordance with the Declaration of Helsinki, the study protocol was approved by the Ethics Committee of the participating hospital on 01/02/2021 and registered at https://www.isrctn.com/(ID ISRCTN11242213).

The cohort included 5,517 consecutive patients with a probable or certain diagnosis of COVID-19, with or without microbiological diagnostic confirmation, who had at least one visit to the emergency department (ED) during the study period. Patients met at least one of the following inclusion criteria: primary and/or secondary diagnosis codes (ICD-10) for clinically diagnosed COVID-19 (30); having a positive RT-PCR. Exclusion criteria included: being under 18 years of age, being admitted to a medicalized hotel and/or having an advanced or terminal disease. The study objectives were evaluated in the study cohort with assessed nutritional status (N=3,402) and all the analyses involving patient stratification according to body mass index (BMI) were done for a secondary cohort of 3,371 patients, since the quantitative BMI record was missing in 31 patients. Additionally, the study objectives were analyzed in a sub-cohort of patients aged less than 70 years within the overall cohort, considering N=1,871 patients with an assessed nutritional status and N=1,847 patients with quantitative BMI data.

### 2.2 Study endpoints

The primary endpoint was to calculate the frequency of obesity and obesity-related comorbidities, specifically DM, HT, and CVD among COVID-19 positive patients to further estimate the clinical impact and cost. The secondary objective was to investigate the association of obesity status, obesity degree and obesity-related comorbidities with the clinical outcomes of hospitalization, ICU hospitalization, need for IVM and mortality, and the outcome of healthcare cost based on resource utilization. Adjustments for secondary endpoints were based on the results of primary endpoint, including adjusting for demographic variables and the presence of DM, HT and CVD at the time of ED visit.

### 2.3 Data sources and variables

Demographic and clinical data used for this analysis were extracted from the BDCLIN_HCSC_COVID-19 database of the participating hospital, which integrates information from several hospital departments including the ED, microbiology department, and hospital pharmacy. CVD included coronary heart disease, stroke, hypertensive heart disease, inflammatory heart disease, rheumatic heart disease, transient ischemic attack, and other cardiovascular diseases such as tumors, cardiomyopathy, and heart valve diseases. BMI was extracted from the electronic health records at specialized care and/or primary care, in order to have the most updated measurement in each case. For each patient, the BMI value closest to the ED visit was chosen. Records older than 10 years were discarded. Based on their BMI (kg/m^2^) records, patients were classified into non-obesity and obesity groups (this one additionally subclassified into obesity I, II or III), according to the World Health Organization (WHO) and the Centers for Disease Control and Prevention (CDC) criteria (Obesity: BMI≥30; Obesity I: BMI=30-34.9; Obesity II: BMI=35-39.9; Obesity III: BMI≥40). Data related to ED and hospital stay (conventional hospitalization and ICU) were obtained from the Minimum Basic Hospital Discharge Data Set 2020 and the ICU database. The need for IMV was collected using the related ICD-10-PCS. Mortality outcome was assessed using two different variables 1) 30-day mortality (from the first contact with the hospital to day 30) and in-hospital mortality (from the first contact with the hospital to the event of death occurring at any time during the patient’s admission), analyzed both as a dichotomous yes/no variables. Thirty-day mortality was also analyzed as time to event (survival) using Kaplan-Meier analysis. Regarding cost variables, the categories of health expenditure considered were days in ED, length of stay in conventional hospitalization, length of stay in ICU, pharmacological treatment with a high economic impact, and prolonged IMV (having undergone tracheostomy procedure with IMV for more than 96 hours). For the length of stay in each inpatient unit, the total per patient was calculated, considering the number of admissions in each inpatient unit and stay. Additional costs of medication and procedures were included in the cost of each corresponding inpatient unit. The unit costs were obtained from the hospital departments and external sources (31). The cumulative cost per patient was calculated as the sum of all cost components, obtained by multiplying the unit cost of each health expenditure category by the patient’s use of the corresponding resource.

### 2.4 Statistical analyses

The analyses were performed in the global cohort and in the subgroup of patients younger than 70 years aiming to decrease the age effect on COVID-19 morbidity and mortality, according to previous research (32, 33). All data processing and analysis were performed using the Statistical Package for the Social Sciences (IBM SPSS Statistics version 26, IBM Corp., Armonk, NY, USA) and Stata 17. All p-values lower than 0.05 were deemed statistically significant.

#### 2.4.1 Analysis of clinical variables

Sociodemographic and clinical characteristics of patients and the use of healthcare resources were analyzed by descriptive statistics. Absolute and relative frequencies were used to describe categorical variables. The Clopper-Pearson exact method was used to calculate confidence intervals for the prevalence of obesity. Quantitative variables were summarized with their mean and standard deviation (SD) and those showing a skewed distribution were summarized with median and interquartile range (IQR). For the comparison of qualitative variables, the Chi-square test or Fisher’s exact test were used, if necessary. Comparisons of means between two independent groups were performed by Student’s t-test if the variables followed a normal distribution, or by the nonparametric Mann-Whitney U test for asymmetric variables. Comparisons of means between more than two independent groups were performed by analysis of variance (ANOVA), or by the nonparametric Kruskal-Wallis test for asymmetric variables. A multivariate logistic regression analysis was performed to assess the risk of hospitalization, risk of ICU admission, need for IMV and risk of mortality associated with obesity diagnosis, obesity degree and obesity-related comorbidities categories. For each of the outcome variables, crude odds ratios (OR) adjusted for age, sex, presence of HT, DM, and history of CVD were calculated. In each table, the reference category is specified. The relationship between obesity and 30-day mortality was also explored by survival analysis using the Kaplan-Meier method, where the log-rank test was used to compare survival functions. Cox regression models were used to obtain the crude and adjusted (age, sex, presence of HT, DM, and history of CVD) effect of obesity on the 30-day mortality rate. The hazard ratio (HR) is presented as a measure of effect.

#### 2.4.2 Analysis of economic variables

For the economic impact assessment, multivariate regression models were performed with log(cost) as the dependent variable, adjusted for covariates that could influence the cost (i.e., age, sex, country of birth, and comorbidities). To correct the selection bias caused by missing values of the variable of interest nutritional status, a missing data multiple imputation statistical procedure was designed (see **Supplementary Material Tables S5, S6**). An individual obesity status has been imputed to each patient with an unrecorded diagnosis of obesity, estimated from the information available in the sample. The procedure, which is applied to the whole sample and for the specific group of patients under 70 years of age, is as follows: A stratified random sample with replacement, of size 1,000 for the total sample and 800 for the under-70s, is drawn from the population of patients with known obesity status. It is stratified according to the quartiles of the age distribution of the missing group, so that its age composition is similar. A binary probit model (**Table S6**) of obesity is estimated whose predictors are age and its square and the dummies of sex, CVD, DM and HT, Latin American origin and other countries. The estimated equation is used to predict the probability of obesity of the missing patients, and the binary variable obese/non-obese is defined with a cut-off point of 0.5 on the predicted probability. This process (1-2) is repeated 100 times and the results of the 100 samples are recorded to obtain 100 cost estimates for both patients with and without obesity and their corresponding standard deviations.

## 3 Results

### 3.1 Study population

**Figure 1:**
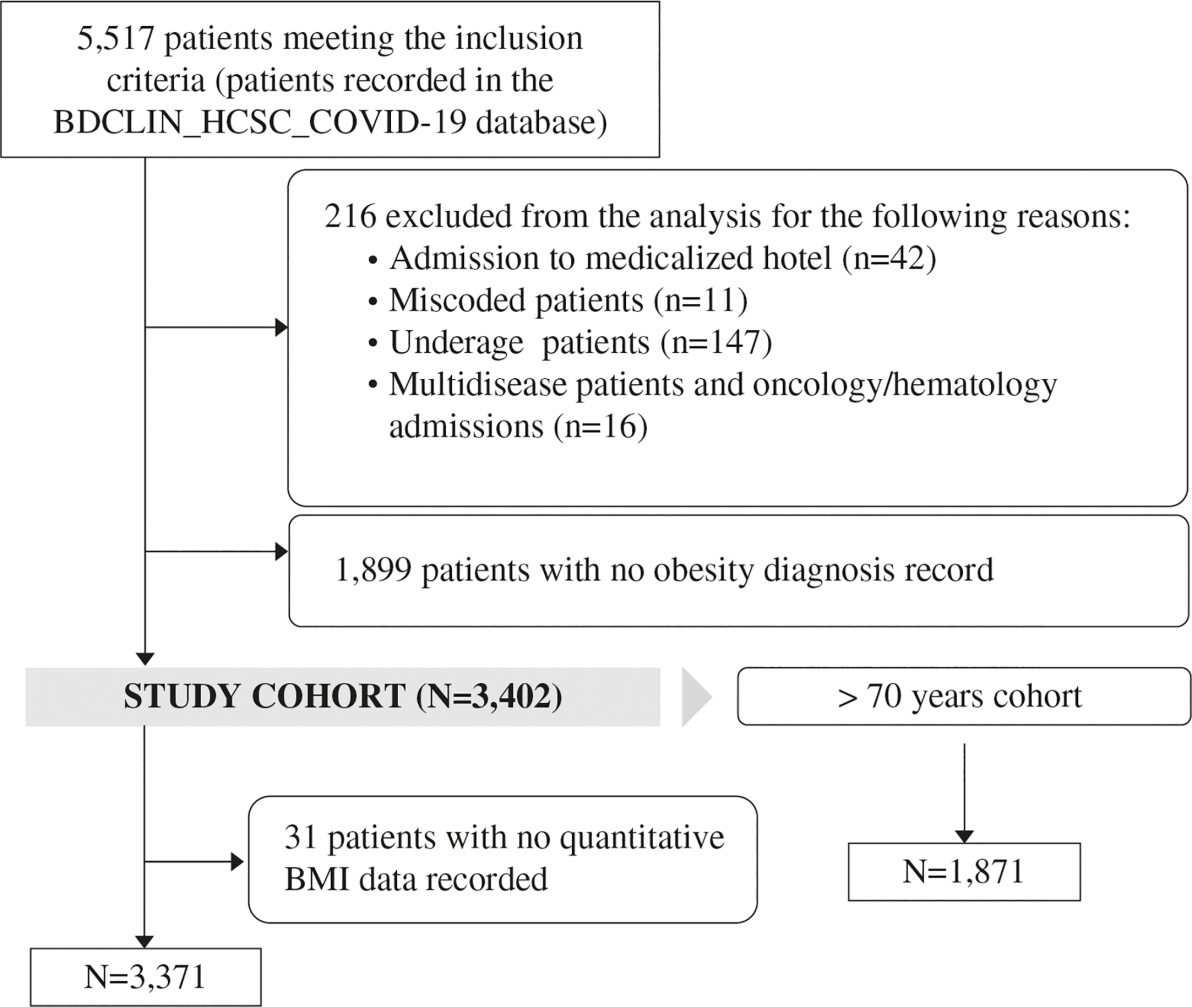
Patient inclusion flowchart.

Between March 1, 2020 and June 30, 2020, 5,517 patients meeting the inclusion criteria were identified from the BDCLIN_HCSC_COVID-19 database, of whom 216 patients were excluded consensually from the analysis to minimize the effect of confounding factors, and 1,899 were excluded due to absence of BMI record. Therefore, 3,402 patients were evaluable for the study analysis (**Figure 1**). **Table 1** shows the baseline demographic and clinical characteristics of patients included in the study cohort and in the subgroup of patients aged <70 years. Patient’s baseline characteristics according to obesity degree and the presence or absence of obesity-related comorbidities are shown in **Supplementary Tables S1**, **S4**, respectively.

**Table 1:** Demographic and main clinical characteristics of the study cohort.

Baseline characteristics	Overall population (N=3,402)	Population <70 years old (N=1,870)	*p* value
Age (years), mean ± SD,	64.7 ± 18.8	50.4 ± 12.4	<0.001
Female sex, n (%)	1,786 (52.5)	1,058 (56.6)	0.0045
BMI (kg/m^2^), mean ± SD*	28.3 ± 5.2	28.6 ± 5.6	0.055
Obesity degree*			<0.001
Obesity I, % (95% CI)	23.1 (21.7-24.6)	23.0 (23.1-24.9)	
Obesity II, % (95% CI)	6.9 (6.1-7.8)	8.0 (6.8-9.3)	
Obesity III, % (95% CI)	2.8 (2.3-3.4)	4.0 (3.1-4.9)	
Origin, n (%)			<0.001
Spanish	2,535 (74.6)	1,069 (57.1)	
Latin American	709 (20.8)	684 (36.6)	
Other countries	154 (4.5)	118 (6.3)	
Emergency room stay but no admission, n (%)	1,362 (40.0)	1,019 (54.5)	<0.001
Length of stay in ED (days/h), mean ± SD	1.7 ± 0.7	1.7 ± 0.7	<0.001
Length of stay in ED (days/h), median (IQR)	2 (1-2)	2 (1-2)	<0.001
Inpatient hospitalization (at least one admission), n (%)	2,040 (60.0)	852 (45.5)	<0.001
Length of inpatient hospitalization (days), median (IQR)	8 (4-16)	7 (4-5)	0.017
Readmission within 30 days of discharge from the first admission, n (%)	77 (2.3)	31 (1.7)	0.137
ICU admission, n (%)	153 (4.5)	121 (6.5)	0.002
Mechanical ventilation, n (%)	137 (4.0)	109 (5.8)	0.003

BMI, Body mass index; CI, confidence interval; ED, emergency department; ICU, intensive care unit; IQR, interquartile range; SD, standard deviation.

*The sample with available BMI records was 3,371 patients in overall population and 1847 in population <70 years old.

We found a 33.4% (95% CI, 31.8-35.0) prevalence of obesity among the evaluable COVID-19 patients. The rate by sex was 34.2% (32.0-36.5%) in females and 32.5% (30.2-34.8%) in males, with no significant differences between groups (*p* = 0.287). Patients with obesity were significantly younger than patients without obesity (63.79 ± 17.35 years vs. 65.18 ± 19.57 years; *p* = 0.035). Regarding the country of origin, the prevalence of obesity was significantly lower in Spanish patients (31.4%) than in patients from Latin America (39.8%) and other countries (37.0%) (*p* < 0.001).

In the population aged <70 years, the rate of obesity was significantly higher than in patients older than 70 years (35.7%, 95% CI, 33.5-37.9 vs. 30.6%, 95% CI, 28.3-32.9), (*p* = 0.002).

**Figure 2:**
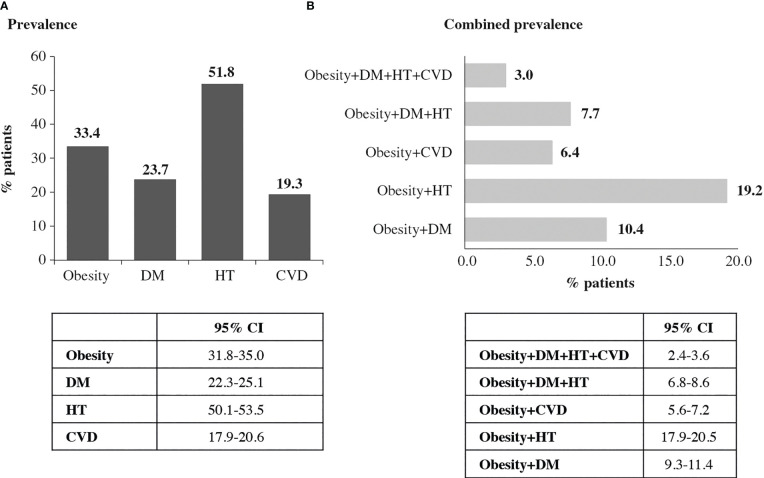
Obesity and obesity-related comorbidities prevalence (N=3,402). **(A)** Individual obesity and obesity-related comorbidities prevalence. **(B)** Combined obesity and obesity-related comorbidities prevalence.

Several evaluable patients (40.2%) did not show any of the studied comorbidities (DM, HT and CVD). A total of 32.2% of patients had at least one comorbidity, and 20.4% and 7.3% had two or three comorbidities, respectively. **Figure 2** shows the prevalence of obesity, independent comorbidities, and combined prevalence, defined as the coexistence of a diagnosed obesity status and at least one of the obesity-related comorbidities already defined in the study cohort. The most predominant combination was obesity and HT (19.2%). There were significant differences in the obesity rate based on the diagnosis of DM (43.6% vs 30.2%, *p* < 0.001) and HT (37.1% vs 29.5%, *p* < 0.001), but not with regard to CVD (33.1% vs 33.5%, p = 0.874). In the subgroup aged <70 years, 62.4% of patients had no comorbidities, 26.4% had at least one comorbidity, and 8.9% and 2.2% had 2 and 3 comorbidities, respectively. The prevalence was 14.5% DM, 30.0% HT and 6.4% CVD

### 3.2 Hospitalization and ICU admission

**Table 2** shows the profile of patients in each inpatient unit: ED without admission, conventional hospitalization, and ICU. Overall, 60% of patients required hospitalization. Global comparisons among three groups were always significant for each variable (*p* < 0.001) Obesity was more prevalent in patients that required hospitalization (35.6%, 95% CI, 33.4-37.8) than in patients only visiting the ED (29.4%, 95% CI, 27.0-31.9; *p* < 0.001). The prevalence of obesity was also significantly higher in patients requiring ICU admission (41.2%, 95% CI, 33.3-49.4) than in non-admitted patients (35.6% 95% CI 33.4-37.8; *p* = 0.003). There were statistically significant differences in the distribution of obesity categories between the No admission and non-ICU admission groups (*p* = 0.02), between the No admission and Inpatient with ICU admission groups (*p* < 0.001), and between the Inpatient without ICU and Inpatient with ICU admission groups (*p* < 0.001) (**Table 2**).

**Table 2:** Profile of patients with COVID-19 according to inpatient unit.

Variable	No hospital admission (N=1,362)	Inpatient, non-ICU (N=1,887)	Inpatient, ICU admission (N=153)	*p* value *(No hospital admission vs. Inpatient, no ICU)*	*p* value *(Inpatient, no ICU vs. Inpatient ICU-admission)*	*p* value *(No hospital admission vs. Inpatient, no ICU)*
Age (years), mean ± SD	56.04 ± 18.74	71.31 ± 16.70	60.60 ± 11.17	**<0.001**	**0.002**	**<0.001**
Female sex, n (%)	815 (59.8)	923 (48.9)	48 (31.4)	**<0.001**	**<0.001**	**<0.001**
Non-obesity, % (95% CI)	70.6 (68.1-73.0)	65.4 (63.2-67.6)	58.8 (50.6-66.7)	<0.001	0.003	0.168
Obesity, % (95% CI)	29.4 (27.0-31.9)	35.6 (33.4-37.8)	41.2 (33.3-49.4)			
Obesity degree				0.020	**<0.001**	**<0.001**
Obesity I, % (95% CI)	20.7 (18.6-23.0)	24.8 (22.8-26.8)	24.2 (17.6-31.8)			
Obesity II, % (95% CI)	6.3 (5.1-7.7)	7.2 (6.1-8.5)	7.8 (4.1-31.8)			
Obesity III, % (95% CI)	2.4 (1.6-3.3)	2.6 (1.9-3.4)	9.2 (5.1-14.9)			
Obesity without obesity-related comorbidity, % (95% CI)	14.1 (12.3-16.1)	8.6 (7.4-10.0)	14.4 (9.2-21.0)	**<0.001**	**0.001**	0.054
Obesity with obesity-related comorbidity, % (95% CI)	15.3 (13.5-17.4)	27.0 (25.0-29.0)	26.8 (20.0-34.5)			

CI, confidence interval; ICU, intensive care unit; SD, standard deviation.

All variables were compared using the Chi-square test, except for the variable “age” which was compared in multiple comparison with ANOVA test.

Bold values stand for statistically significant differences.

### 3.3 Risk of hospitalization, ICU admission, IMV, and mortality

There were significant differences in the need for hospitalization according to obesity status (*p* < 0.001) and obesity categories (*p* = 0.007). The multivariate logistic regression analysis showed that patients with obesity had a higher risk of hospitalization than patients without obesity (OR [95% CI]=1.46;, [1.24-1.73] *p* < 0.001), and the risk of hospitalization increases with increasing obesity (type 1: OR[95% CI]= 1.28[1.06-1.55], *p* =0.010 < type II: OR[95% CI]= 1.58[1.16-2.15], *p* =0.004 < type III: OR[95% CI]= 2.09[1.31-3.34], *p* =0.002). There were significant differences in the need for ICU admission and IMV according to obesity categories (*p* < 0.001). In addition, patients with type III obesity had a significantly higher risk of ICU admission (OR[95% CI]: 3.30 [1.66-6.53]; *p* = 0.001) and IMV need (OR[95% CI]= 3.98[2.00-7.94] *p* < 0.001) as compared to those without obesity (**Table 3**). There were no significant differences in the need for ICU admission according to obesity condition (*p* = 0.168). There was a significant association between the need for IMV and obesity status (*p* = 0.032); however, no association between exposure and outcome variables was observed.

**Table 3:** Risk of hospitalization, ICU admission, IMV and mortality based on the presence of obesity and obesity degree in the overall population.

Overall population (N=3,402)	Obesity		Obesity I		Obesity II		Obesity III	
	OR (95% CI)	*p* value	OR (95% CI)	*p* value	OR (95% CI)	*p* value	OR (95% CI)	*p* value
Hospitalization	**1.46 (1.24-1.73)**	**<0.001**	**1.28 (1.06-1.55)**	**0.010**	**1.58 (1.16-2.15)**	**0.004**	**2.09 (1.31-3.34)**	**0.002**
ICU admission	1.08 (0.76-1.53)	0.674	0.99 (0.66-1.49)	0.970	0.94 (0.49-1.80)	0.848	**3.30 (1.66-6.53)**	**0.001**
IMV	1.26 (0.87-1.81)	0.219	1.13 (0.74-1.73)	0.574	1.14 (0.59-2.21)	0.700	**3.98 (2.00-7.94)**	**<0.001**
Mortality (30-day)	1.03 (0.80-1.33)	0.834	0.93 (0.70-1.24)	0.617	1.26 (0.79-2.02)	0.352	1.56 (0.72-3.40)	0.261
Mortality (in-hospital)	0.99 (0.78-1.27)	0.961	0.89 (0.67-1.18)	0.425	1.15 (0.72-1.83)	0.569	1.56 (0.75-3.28)	0.238

CI, confidence Interval; ICU, intensive care unit; IMV, invasive medical ventilation; OR, Odds ratio.

Reference category: non-obesity. Results adjusted for age, sex, and comorbidities (DM, HT, and CVD).

The logistic regression analysis for the variables “ICU admission”, “IMV” and “mortality” were calculated in the total number of patients requiring hospital admission (N=2,040).

Bold values stand for statistically significant differences.

Results of the analyses on the combination of obesity status and the presence of obesity-related comorbidities are shown in **Table 4**. There were significant differences in the need for hospitalization according to comorbidity categories (*p* < 0.001). Patients with comorbidities (obese and non-obese) showed a higher risk of hospitalization as compared to patients without obesity and without any comorbidity. There were significant differences in the need for ICU admission (*p* = 0.018) and the need for IMV (p = 0.005) according to comorbidity categories; however, no association between exposure and outcome variables was observed. According to the Kaplan-Meier analysis, survival was significantly longer in patients without comorbidities, regardless of the presence of obesity (*p* < 0.001). However, the Cox regression model adjusting for age and sex showed that there was no association between comorbidity categories and survival.

**Table 4:** Risk of hospitalization, ICU admission, IMV and mortality based on the presence of comorbidities in the overall population.

Overall population (N=3,402)	Non-obesity with obesity-related comorbidity		Obesity without obesity-related comorbidity		Obesity with obesity-related comorbidity	
	OR (95% CI)	*p* value	OR (95% CI)	*p* value	OR (95% CI)	*p* value
Hospitalization	1.85 (1.49-2.28)	<0.001	1.67 (1.30-2.14)	<0.001	2.29 (1.84-2.87)	<0.001
ICU admission	1.31 (0.78-2.21)	0.314	1.15 (0.64-2.05)	0.638	1.34 (0.80-2.25)	0.265
IMV	1.19 (0.69-2.08)	0.532	1.25 (0.69-2.28)	0.459	1.49 (0.87-2.54)	0.146
Mortality (30-day)	1.12 (0.74-1.69)	0.592	0.76 (0.37-1.58)	0.468	1.22 (0.79-1.87)	0.353
Mortality (in-hospital)	0.99 (0.68-1.47)	0.985	0.83 (0.44-1.58)	0.573	1.03 (0.69-1.54)	0.873

CI, confidence Interval; ICU, intensive care unit; IMV, invasive medical ventilation; OR, Odds ratio.

Reference categories: non-obesity without any obesity-related comorbidity. Results adjusted for age and sex Obesity-related comorbidities have been previously defined in this text as diabetes mellitus, arterial hypertension, and cardiovascular disease. The logistic regression analysis for the variables “ICU admission”, “IMV” and “mortality” were calculated in the total number of patients requiring hospital admission (N=2,040).

*Post hoc* analyses in the subgroup of patients aged <70 showed similar results (**Supplementary Materials Tables S2, 3**). No significant associations were found with any of the outcome variables in patients over 70 years of age.

### 3.4 Mortality

The in-hospital mortality rate was significantly higher in patients without obesity than in those with obesity (22.2% vs. 17.8%; *p*=0.018). There were also significant differences in 30-day mortality from the first ED contact within the cohort of hospitalized patients (N=2,040) according to obesity status, with a higher mortality rate in non-obese patients (21.0% vs. 17.0%; *p* = 0.029). The regression analysis showed no differences in the risk of in-hospital or 30-day mortality in patients with and without obesity, and neither across obesity degrees (**Table 3**). The Kaplan-Meier analysis showed a significantly higher survival in patients with obesity compared with non-obese patients (*p* = 0.032). However, neither of the groups reached the median OS at day 30 (**Supplementary Material Figure S1**). The results of the Cox regression model adjusting for age, sex and comorbidities show that there was no association between obesity status and overall survival (HR=1.05; 95% CI 0.84-1.30; *p*=0.669). The median 30-day OS was not reached in any obesity category (**Supplementary Material Figure S2**), and there were no significant differences in survival according to obesity categories (*p* = 0.329) and no association between obesity categories and survival was found.

Among patients under 70 years of age, no significant association was found with the mortality outcomes based on obesity status or categories (**Table S2**). The median 30-day OS was not reached in any of the groups (obesity vs. non-obesity) and no statistically significant differences were found (*p* = 0.365). Similarly, no obesity category reached the median 30-day OS and no statistically significant differences were found among categories (*p* = 0.329; **Supplementary Material Figures S3, 4**). The results of the Cox regression model adjusting for age, sex and comorbidities showed no association between obesity categories and survival (HR=1.31; CI 95% 0.46-3.70; *p* = 0.612).

### 3.5 Healthcare resource use and cost

The total average cost per patient for patients with obesity (N=3,402) was €10,805, substantially higher than the average cost per patient in patients without obesity (€8,418.30, p = 0.007). **Table 5** shows the mean cost according to obesity status in the overall population and patients under 70 years, including cost for each healthcare resource use and the percentage excess cost associated with obesity status. The excess cost of obesity in the study cohort was 28.4%, reaching 56.5% in patients under 70 years of age. When the data was imputed in those patients with no available BMI record, the magnitude of the obesity excess cost is even higher (**Table S6**).

**Table 5:** Average costs per COVID-19 patient according to obesity status.

	Overall population (N=3,402)			Population <70 years old (N=1,871)		
	Average cost Non-obese (€)	Average cost Obese (€)	Obesity excess cost (%)	Average cost Non-obese (€)	Average cost Obese (€)	Obesity excess cost (%)
Emergency department	316.9	329.6	4.0	301.0	321.6	6.8
Hospitalization	5,790.0	6,674.0	15.3	3,443.0	5,379.0	56.2
ICU admission	1,686.0	2,738.0	62.4	2,428.0	3,818.0	57.2
Medication	38.0	67.7	78.2	50.9	98.2	92.9
Tracheostomy procedures + IMV >96 h	650.7	995.4	53.0	846.7	1434.3	69.4
Total	8,418.3	10,805.0	28.4	7,070.0	11,051.0	56.3

ICU, intensive care unit; IMV, invasive mechanical ventilation.

**Figure 3:**
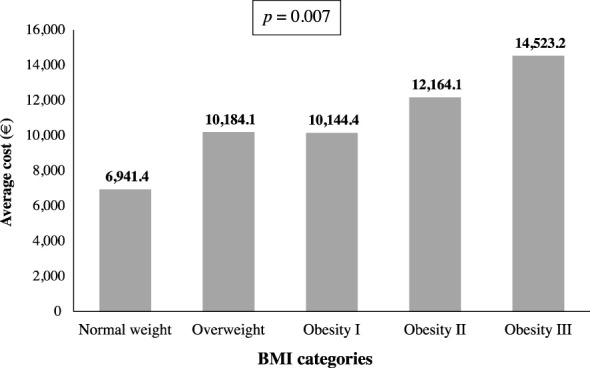
Average cost per patient according to BMI categories (N=3,371).

**Figure 4:**
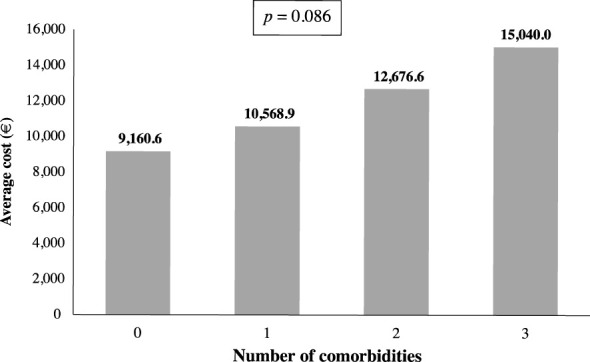
Average cost per patient according to number of comorbidities (N=3,402).

There were significant differences in the mean total cost by BMI category (p=0.007), the average cost increased with obesity degree (**Figure 3**). The lowest cost corresponds to the normal weight category (€ 6,964.42), while the highest cost corresponds to the grade III obesity category (€ 14,523.22). Moreover, the average cost per patient rises with the combination of obesity and two comorbidities (€ 12,676.61) or three OAC (€ 15,039.97) (**Figure 4**).

## 4 Discussion

The OBESITY-COVID study evaluated the prevalence of obesity and its associated comorbidities in a large cohort of patients attending the emergency service of a Spanish hospital during the first wave of the COVID-19 pandemic. Our results show a high prevalence of obesity (33.4%) and comorbidities (HT: 51.8%, DM: 23.7% and CVD: 19.3%) among COVID-19 patients in the participating center, an excess cost burden of obesity of 28%, and a higher risk of hospital admission, ICU admission and need for IMV for patients with obesity. However, we did not find a significant association between obesity and COVID-19 mortality. To our knowledge, this is the first Spanish study specifically evaluating the rate and association of obesity and comorbidities with costs in a large cohort of patients.

Our results confirmed obesity as a robust predictor of critical outcomes in COVID-19 patients (21, 24, 34–36). We found a higher rate of obesity in those patients requiring hospitalization (35.6%), and even higher in patients requiring ICU admission (41.2%). The odds of being hospitalized were significantly higher when the patients had obesity, and they increased with BMI, reproducing what has been described in large cohorts (37). Patients with BMI over 40 kg/m^2^ had a higher risk of hospitalization, ICU admission and to receive IMV, regardless of age, sex and diagnosis of comorbidities. This increased odds for ICU admission in grade III obesity, without being significant for obese status, has been described in similar studies (24, 38). Previous studies used a cut-off point around 70 years of age to stratify the sample, finding a greater association in the need for hospitalization among patients under 65 years (37) or under 70 years of age (39). Other studies have found a more significant association when stratifying the sample below 60 years (40) or even 50 years (38, 41). Our study also describes a higher association between obesity and obesity degrees with the need for hospitalization in those under 70 years of age, with no significant association found in the age range over 70 years. In the need for ICU admission and the need for IMV, no differences were observed stratifying by age, which should be analysed considering that the average age of the patient admitted to the ICU is 60.6 years.

Both in-hospital mortality and 30-day mortality rates were significantly lower in patients with obesity, and we found no association between mortality and obesity in the multivariate analysis. These findings are in line with the results obtained in three relevant studies. One of them is the systematic review and meta-analysis of 120 studies, which could not find an association between obesity status and mortality in COVID-19 patients (26). Moreover, the HOPE-COVID-19 retrospective cohort registry, evaluating a similar-sized cohort of patients, suggested that BMI was not a mortality predictor (15). Finally, a study performed in Madrid with a cohort of patients receiving antiviral treatment for COVID-19 reported a relationship between obesity and the development of acute respiratory distress syndrome, but not between obesity and mortality (18). Nevertheless, some other large cohort studies and meta-analyses demonstrated a reliable link between obesity and mortality (12, 17, 20–25). Based on these controversial results, the potential association between obesity and mortality in COVID-19 patients deserves to be studied in greater depth.

This study showed a rate of obesity of 33.4%, which increases to 35.7% in patients younger than 70 years old. This percentage is substantially higher than the obesity prevalence found in the SIESTA cohort, representing almost all the Spanish territory (14.3%) (20), and in the study by Rodriguez-Gonzalez et al. (15.1%) (18), which included a cohort from the same region as ours. This might be due to differences in patient population, since the SIESTA cohort consisted of a random sample of patients clinically or microbiologically diagnosed with COVID-19, and many were asymptomatic patients not attending the ED, while all patients included in our study came from the ED. The obesity prevalence found in our cohort is within the range of that reported in larger studies, ranging from 25 to 42% (23, 25, 26, 29, 34, 35, 42). The obesity rate by sex and age range is comparable to that reported in the ENRICA study (43),which shows an increase in the prevalence of obesity over 65 years of age (44), suggesting that our cohort is representative of the Spanish population and that the results of our study could be aligned with it.

The DM rate found in our study (23.7%) is slightly higher than the range of prevalence previously reported for this condition in COVID-19 patients (17-22%) (18, 20, 26, 35, 42). Similarly, the HT rate (51.8%) observed in our study was slightly higher than that found in prior reports ranging from 32 to 45% (18, 20, 26, 35, 42). CVD prevalence in COVID-19 patients showed variability between studies (13% (35), 16% (26) and 31.4% (18)). Our data remains within this range (19.3%). In the subgroup of patients under 70 years of age, the prevalence of comorbidities was significantly lower than in the overall cohort.

We found that patients with comorbidities had a higher risk of hospitalization and those without comorbidities showed the highest OS, regardless of their BMI, in line with prior reports (14, 26, 35, 45). However, Spanish investigators could not find an association between the presence of DM and CVD and mortality in the SIESTA cohort (20).

Some studies carried out in Spain had evaluated resource utilization in cohorts of COVID-19 patients at different stages of the disease (18, 27) but, to our knowledge, none of the studies conducted so far has undertaken a comparative analysis between patients with and without obesity. As expected, our data show that the cost per patient was significantly higher in patients with obesity compared to patients without obesity, with an excess cost of 28.4% in the overall cohort and 56.5% in patients under 70 years of age. Additionally, there is a significant increase in the average cost per patient when the obesity degree increases, with the highest excess cost in subjects with grade III obesity. Furthermore, we observed a rising trend in the average cost when the number of comorbidities increases, but it was not statistically significant, likely because the number of patients decreases as the number of comorbidities increases.

This increase in health expenditure in patients with obesity has been previously reported. In a cohort of patients from 273 hospitals located in the U.S., overweight and obesity were associated with higher economic cost of inpatient care, with the highest excess cost in patients with BMI over 45kg/m^2^ (46). This increased cost associated with obesity status is also reported in a European study evaluating the costs of COVID-19 associated with obesity in the first 6 months of the pandemic (29). According to this study, the average costs for patients without obesity are aligned with ours (taking into account differences in the study timeframe, 6 months vs. 3 months), but the average cost for patients with obesity is substantially higher. Differences with this European study might come from differences in the study design (cost model estimation vs. single-center observational study). The economic excess cost associated with obesity, along with the increased risk of critical outcomes, reinforce the need to address obesity treatment as a health priority.

This investigation has some limitations. First, participation of a single center located in Madrid, which was one of the most severely stricken areas during the COVID-19 pandemic in Spain, may entail bias. Data such as risk for hospitalization, ICU admission and in-hospital mortality in obese patients cannot be extrapolated to other countries or other regions in Spain. Secondly, the timeframe in which this study was conducted corresponds to the pandemic first wave, from March to June 2020, a period in which emergency and inpatient activity, and especially ICU occupancy, were determined by the high occupancy demand of Madrid’s community hospitals (46). Despite its limitations, this study provides valuable data from a large cohort of Spanish patients during the most critical period of the COVID-19 pandemic, assessing clinical and economic aspects of the disease.

We must consider these results carefully due to the rapid evolution of the pandemic since the outbreak at the end of 2019 to the current situation in 2023. The virus strain has been evolving and changing its virulence, the standard of care has changed according to scientific evidence and almost all of the population is fully vaccinated, therefore this must be borne in mind when comparing hospitalization, ICU admission and mortality rates as well as the cost analysis from 2020 to the present.

In conclusion, this study suggests a strong association between obesity and inpatient hospitalization, ICU admission, need for IMV in COVID-19 patients and economic excess cost. The risk for adverse outcomes and cost increases with obesity degree and the presence of comorbidities, regardless of obesity status. Likewise, obesity has been associated with poorer clinical outcomes and higher costs in other pandemics that occurred in this century or other noninfectious diseases, therefore we must promote the management of obesity as a health priority

## Data availability statement

The original contributions presented in the study are included in the article/**Supplementary Material**. Further inquiries can be directed to the corresponding author.

## Ethics statement

The studies involving human participants were reviewed and approved by Hospital Clínico San Carlos Ethics Committee. Written informed consent for participation was not required for this study in accordance with the national legislation and the institutional requirements.

## Author contributions

T-EM, LM, R-HM, MV and PF contributed to conceptualization of the study; T-EM, F-FM. and GB contributed with the study methodology; F-FM, GB, B-PP and SC performed the statistical analysis; T-EM, R-HM, MV, PF and LM conducted the investigation; T-EM, NA, G-PC participated in resource searching; T-EM, NA, G-PC contributed to data curation; T-EM, MV, and R-HM wrote the original draft; T-EM, R-HM, LM participated in the supervision; T-EM, LM, R-HM, MV, PF, F-FM, GB and SC contributed to manuscript revision. All authors contributed to the article and approved the submitted version.
