# Supplementary material for: Clinical and economic impact of COVID-19 on people with obesity in a Spanish cohort during the first pandemic peak
Source: Front Endocrinol (Lausanne). 2023 May 26;14:1146517. doi: 10.3389/fendo.2023.1146517 (PMC10278591; doi:10.3389/fendo.2023.1146517)

Supplementary Material

Article Title

Torrego-Ellacuría M^1,2^, Rubio-Herrera MA^2,3^*, González López-Valcárcel B^4^, Fuentes-Ferrer ME^5,6^, Martín V^7^, Poyato F^7^, Barber-Pérez P^4^, Santucci C^4,8^, Nuñez A^9^, González-Pérez C^1,10^, Luaces M^1^

*** Correspondence:** Miguel Ángel Rubio-Herrera: [marubioh@gmail.com](mailto:marubioh@gmail.com)

**Table S1. Baseline characteristics at emergency admission by obesity degree**

| Baseline characteristics | Non-obesity | Obesity I | Obesity II | Obesity III |
| --- | --- | --- | --- | --- |
| Age (years), mean±SD | 65.18±19.57 | 65.55±17.13 | 61.16±18.26 | 57.24±15.79 |
| Age <70 years, n (%) | 1,203 (65.13) | 424 (23.01) | 147 (7.96) | 73 (3.95) |
| Age ≥70 years, n (%) | 1,063 (69.75) | 355 (23.29) | 85 (5.58) | 21 (1.38) |
| Sex |  |  |  |  |
| Male, n (%) | 1,901 (68.23) | 380 (23.76) | 102 (6.37) | 26 (1.6) |
| Female, n (%) | 1,175 (66.30) | 399 (22.52) | 130 (7.33) | 68 (3.84) |
| BMI (kg/m^2^), mean±SD | 25.45 ± 2.99 | 32.09 ± 1.42 | 36.99 ± 1.40 | 43.40 ± 3.14 |
| Origin |  |  |  |  |
| Spanish, n (%) | 1,738 (69.18) | 554 (22.05) | 155 (6.17) | 65 (2.58) |
| Latin American, n (%) | 427 (60.82) | 184 (26.21) | 66 (9.40) | 25 (3.56) |
| Other origin, n (%) | 97 (63.39) | 41 (26.79) | 11 (7.19) | 4 (2.61) |
| Comorbidities |  |  |  |  |
| HT, n (%) | 1,109 (63.51) | 448 (25.66) | 134 (7.67) | 55 (3.15) |
| DM, n (%) | 455 (57.16) | 240 (30.15) | 66 (8.29) | 35 (4.39) |
| CVD, n (%) | 438 (67.48) | 163 (25.11) | 35 (5.39) | 13 (2.0) |

BMI; body mass index; CI, confidence interval; CVD; cardiovascular disease; DM, diabetes mellitus; HT, hypertension; ICU, intensive care unit; SD, standard deviation.

This descriptive analysis was performed in the population of patients with an available BMI record (N =3371).

**Table S2. Risk of hospitalization, ICU admission, invasive mechanical ventilation, and mortality based on the presence of obesity and obesity degree in the population < 70 years old**

| Population <70 years old  (N=1,871) | Obesity | | Obesity I | | Obesity II | | Obesity III | |
| --- | --- | --- | --- | --- | --- | --- | --- | --- |
|  | OR (95% CI) | *p* value | OR (95% CI) | *p* value | OR (95% CI) | *p* value | OR (95% CI) | *p* value |
| Hospitalization | 1.88 (1.55-2.27) | <0.001 | 1.68 (1.5-2.10) | <0.001 | 1.70 (1.20-2.39) | 0.003 | 2.56 (1.57-4.17) | <0.001 |
| ICU admission | 1.05 (0.71-1.55) | 0.795 | 0.96 (0.60-1.52) | 0.847 | 0.91 (0.45-1.85) | 0.792 | 2.70 (1.37-5.33) | 0.004 |
| Invasive mechanical ventilation | 1.27 (0.85-1.89) | 0.253 | 1.13 (0.69-1.82) | 0.621 | 1.11 (0.54-2.29) | 0.765 | 3.32 (1.67-6.60) | 0.001 |
| Mortality (30-day) | 0.76 (0.42-1.36) | 0.351 | 0.52 (0.24-1.15) | 0.105 | 0.76 (0.26-2.21) | 0.612 | 1.34 (0.45-3.96) | 0.601 |
| Mortality (in-hospital) | 0.78 (0.46-1.30) | 0.335 | 0.61 (0.31-1.18) | 0.141 | 0.58 (0.20-1.67) | 0.312 | 1.31 (0.49-3.49) | 0.591 |

CI, confidence Interval; ICU, intensive care unit; OR; Odds ratio.

*Reference category: non-obesity. Results adjusted for age, sex, and comorbidities (DM, HT, and CVD).*

The logistic regression analysis for the variable “ICU admission”, "invasive mechanical ventilation” and “mortality” were calculated in the total number of patients requiring hospital admission (N=852).

**Table S3. Risk of hospitalization, ICU admission, invasive mechanical ventilation and mortality based on the presence of comorbidities in the population < 70 years old**

| Population <70 years old  (N=1,871) | Non-obesity with **obesity-related** comorbidity | | Obesity without **obesity-related** comorbidity | | Obesity with **obesity-related** comorbidity | |
| --- | --- | --- | --- | --- | --- | --- |
|  | OR (95% CI) | *p* value | OR (95% CI) | *p* value | OR (95% CI) | *p* value |
| Hospitalization | 2.23 (1.74-2.87) | <0.001 | 1.61 (1.24-2.08) | <0.001 | 3.69 (2.83-4.82) | <0.001 |
| ICU admission | 1.84 (1.09-3.10) | 0.021 | 1.26 (0.69-2.29) | 0.450 | 1.49 (0.88-2.51) | 0.135 |
| Invasive mechanical ventilation | 1.66 (0.95-2.91) | 0.076 | 1.39 (0.74-2.59) | 0.305 | 1.74 (1.01-2.98) | 0.045 |
| Mortality (30-day) | 2.99 (1.41-6.35) | 0.004 | 1.04 (0.38-2.86) | 0.943 | 1.56 (0.68-3.55) | 0.292 |
| Mortality (in-hospital) | 1.97 (1.03-3.76) | 0.039 | 0.84 (0.36-1.97) | 0.682 | 1.24 (0.62-2.46) | 0.546 |

CI, confidence Interval; ICU, intensive care unit; OR; Odds ratio.

*Reference categories: non-obesity without any obesity-related comorbidity. Results adjusted for age and sex. Obesity-related comorbidities have been previously defined in this text as diabetes mellitus, arterial hypertension, and cardiovascular disease.*

*The logistic regression analysis for the variables “ICU admission”, “IMV” and “mortality” were calculated in the total number of patients requiring hospital admission (N=852).*

**Table S4.** **Baseline characteristics and clinical outcomes according to presence/absence of obesity and comorbidities**

|  | Non-obesity | | Obesity | |
| --- | --- | --- | --- | --- |
|  | Without **obesity-related** comorbidity | With **obesity-related** comorbidity | Without **obesity-related** comorbidity | With **obesity-related** comorbidity |
| Age (years), mean±SD | 51.50±17.27 | 75.79±13.83 | 50.60±14.63 | 70.34±14.67 |
| Age < 70 years | 837 (44.7) | 366 (19.6) | 331 (17.7) | 337 (18.01) |
| Age ≥ 70 years | 153 (10.0) | 910 (59.7) | 46 (3.0) | 422 (27.7) |
| Sex |  |  |  |  |
| Male | 377 (23.3) | 714 (44.2) | 148 (9.2) | 377 (23.3) |
| Female | 613 (34.3) | 562 (31.5) | 229 (12.8) | 382 (21.4) |
| Origin |  |  |  |  |
| Spanish | 621 (24.5) | 1117 (44.1) | 177 (7.0) | 620 (24.5) |
| Latin American | 308 (43.4) | 119 (16.8) | 173 (24.4) | 109 (15.4) |
| Other origin | 59 (38.3) | 38 (24.7) | 27 (17.5) | 30 (19.48) |
| Emergency room stay but no admission | 611 (63.6) | 350 (36.5) | 192 (14.1) | 209 (15.4) |
| Inpatient hospitalization | 379 (29.0) | 926 (71.0) | 163 (8.6) | 509 (27.1) |
| ICU admission | 35 (38.9) | 55 (61.1) | 22 (14.4) | 41 (26.8) |

N (%) unless otherwise stated. SD: standard deviation; ICU: Intensive Care Unit. *Obesity-related comorbidities have been previously defined in this text as diabetes mellitus, arterial hypertension, and cardiovascular disease.*

**Table S5. Cost estimation of health resource use in overall sample with imputed records**

| Health resources | Overall population with imputed records  (n=5,301) | | | Population <70 years old with imputed records  (n=2,499) | | |
| --- | --- | --- | --- | --- | --- | --- |
|  | **Average cost**  **Non-obese (€)** | **Average cost**  **Obese (€)** | **Obesity excess cost (%)** | **Average cost**  **Non-obese (€)** | **Average cost**  **Obese (€)** | **Obesity excess cost (%)** |
| Emergency department | 304.0 | 330.0 | 8.6 | 290.0 | 320.0 | 10.5 |
| Hospitalization | 4,664.0 | 6,662.0 | 42.8 | 2,843.0 | 5,325.0 | 87.3 |
| ICU hospitalization | 1,024.0 | 2,715.0 | 165.1 | 1,221.0 | 3,634.0 | 197.7 |
| Medication | 30.0 | 66.0 | 120.0 | 35.0 | 94.0 | 169.2 |
| Tracheostomy procedures + IMV >96 h | 359.0 | 987.0 | 174.9 | 426.0 | 1,365.0 | 220.7 |
| Total | 6,381.0 | 10,761.0 | 68.6 | 4,814.0 | 10,739.0 | 123.1 |

Adjusted for selection bias by imputation of missing records for obesity based on probit models. ICU: Intensive Care Unit; IMV: invasive mechanical ventilation

**Table S6. Missing value imputation probit models**

| Variable | Overall cohort (n=5,301) | | Population <70 years old (n=2,499) | |
| --- | --- | --- | --- | --- |
|  | Average estimated coefficient | % of samples in which it is significant | Average estimated coefficient | % of samples in which it is significant |
| Age | 0.05 | 98% | 0.08 | 92% |
| Age (squared) | -0.00048 | 98% | -0.0008 | 79% |
| Male | -0.11 | 27% | -0.0222 | 3% |
| Latin American origin and other countries | 0.16 | 15% | 0.1048 | 6% |
| Spanish | -0.21 | 25% | -0.216 | 22% |
| DM | 0.52 | 99% | 0.7088 | 99% |
| CVD | -0.04 | 3% | -0.6898 | 7% |
| HT | 0.32 | 80% | 0.373 | 82% |
| *R^2^* | 0.0583 |  | 0.0711 |  |

Figure S1. Cumulative 30-day overall survival (obesity vs. non-obesity)


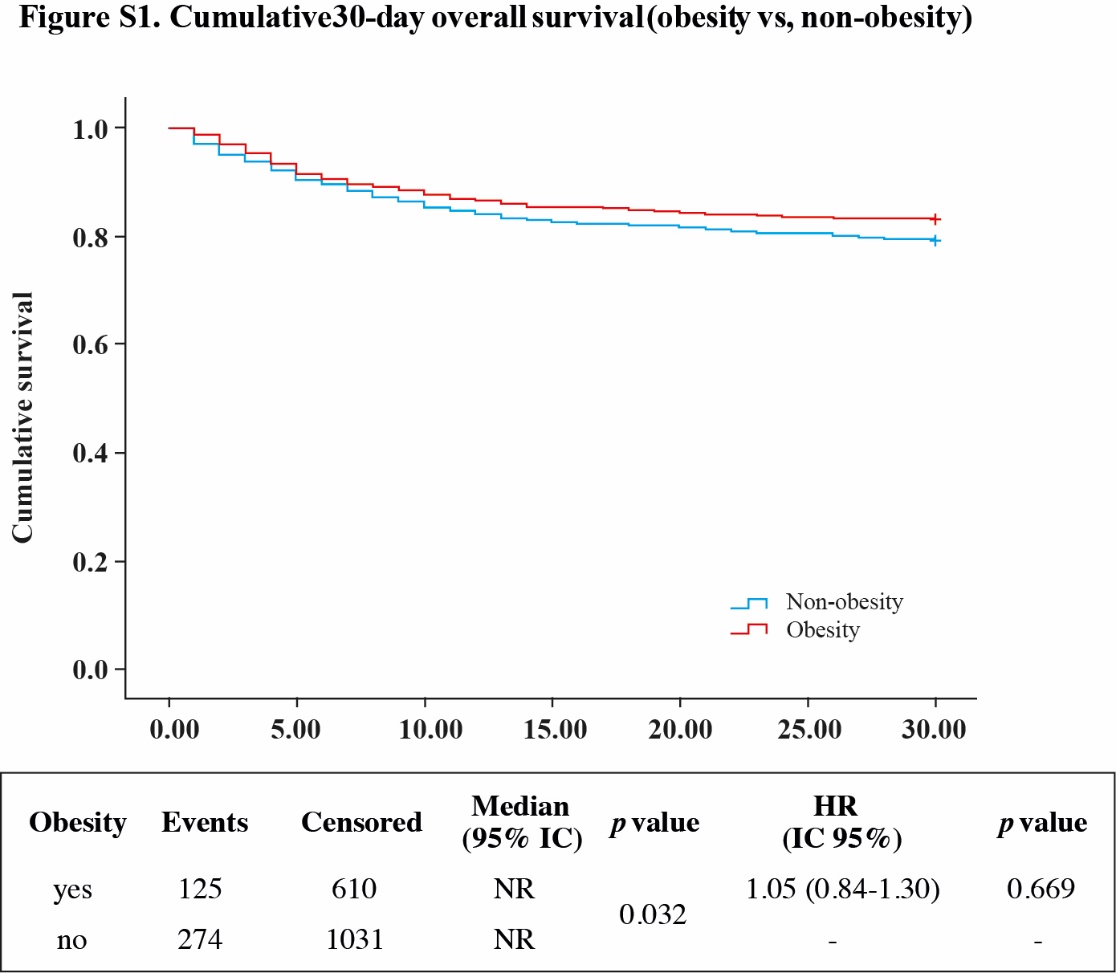


Figure S2. Cumulative 30-day overall survival (obesity vs. non-obesity) by obesity category


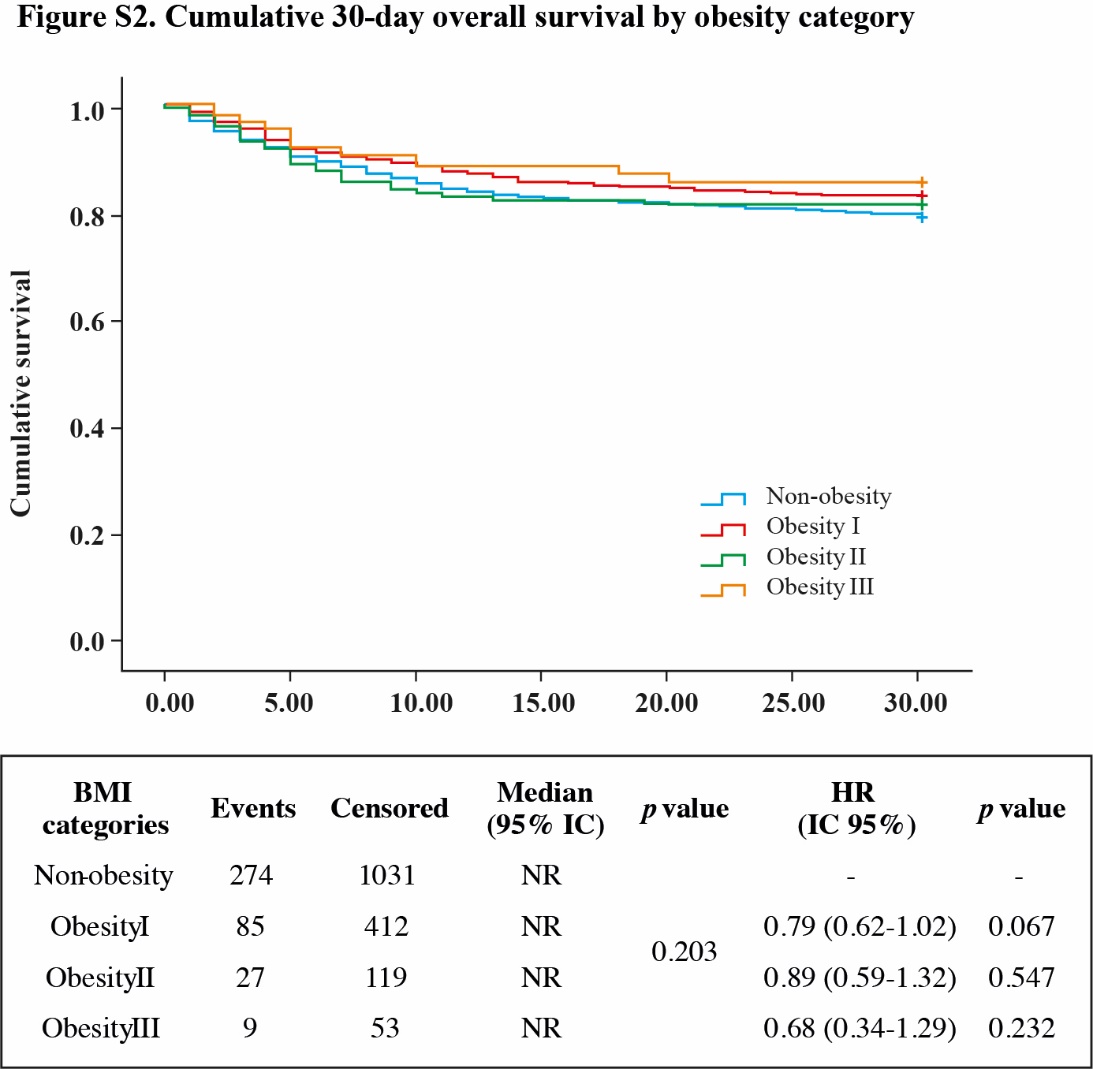


Figure S3. Cumulative 30-day overall survival (obesity vs. non-obesity) in patients < 70 years old


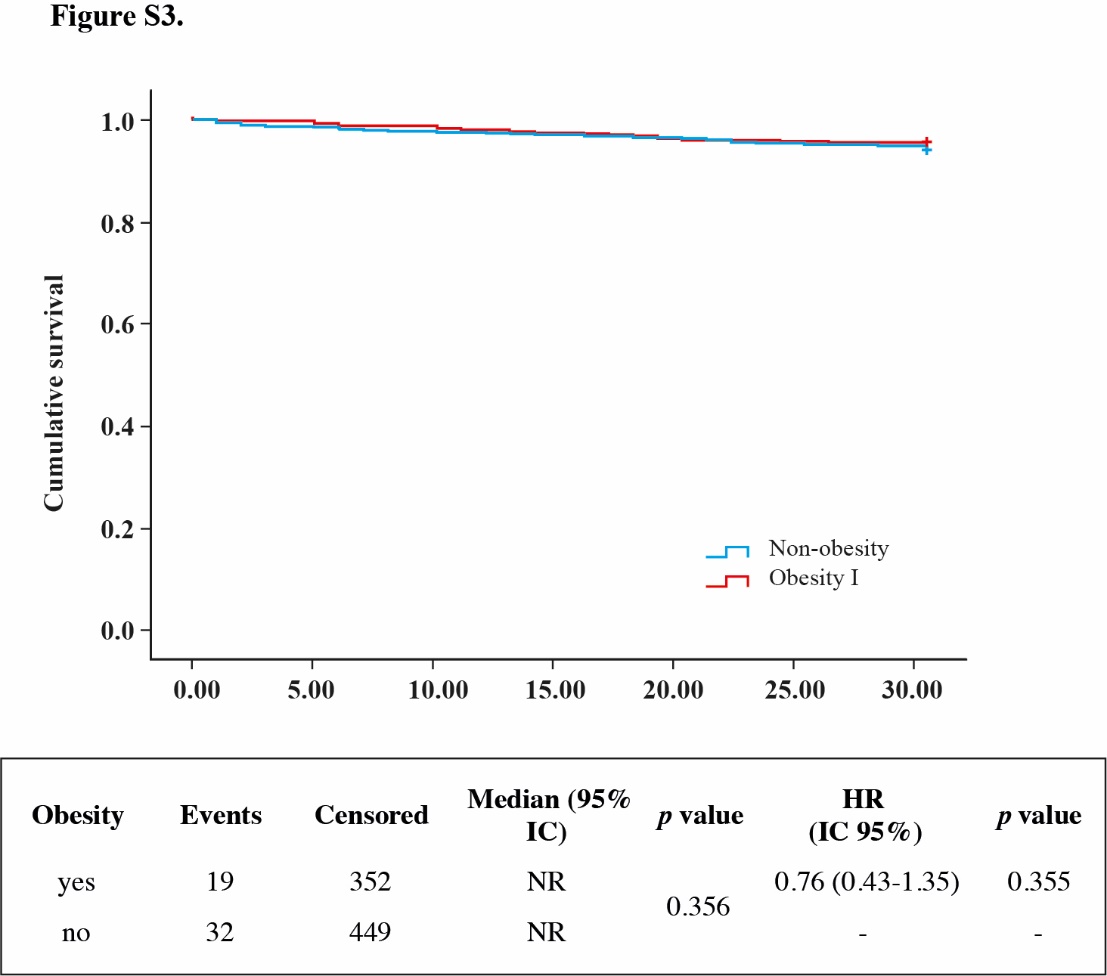


Figure S4. Cumulative 30-day overall survival by obesity category in patients < 70 years old


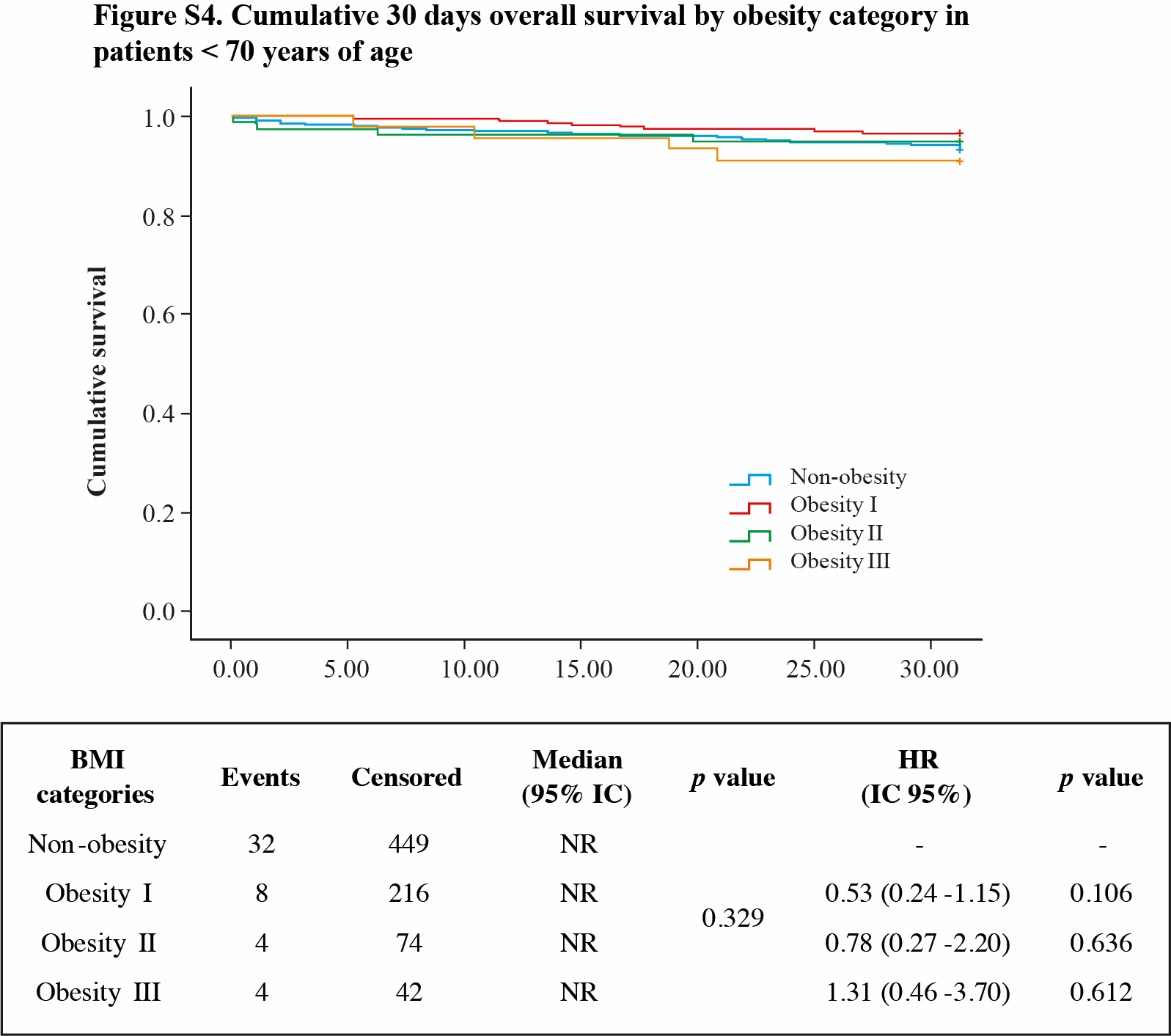

Supplement: Supplementary file 1 [file DataSheet_1.docx]
